# Differentiated impact of social and non-social content in Alexithymia: A facial thermal infrared imaging study

**DOI:** 10.1371/journal.pone.0341921

**Published:** 2026-03-20

**Authors:** Lucas De Zorzi, Eduardo S. Martínez-Velázquez, Jacques Honoré, Henrique Sequeira

**Affiliations:** 1 Sciences Cognitives et Sciences Affectives, Univ. Lille, CNRS, UMR 9193 - SCALab - Sciences Cognitives et Sciences Affectives, F-59000 Lille, France; 2 Laboratory of Psychophysiology and Social Cognition, Faculty of Psychology, Benemérita Universidad Autónoma de Puebla, Puebla, Mexico; 3 Department of Biology, Faculty of Sciences and Technology, Univ. Lille, Lille, France; BSMHFT National Centre for Mental Health, UNITED KINGDOM OF GREAT BRITAIN AND NORTHERN IRELAND

## Abstract

Alexithymia, a difficulty in processing and expressing emotions, is associated with socio-emotional challenges and altered autonomic responses. This raises the question of whether cognitive (CA) and affective (AA) alexithymia can be differentiated based on parasympathetic and sympathetic indices. This study explores the reactivity of CA and AA individuals to social and non-social emotional stimuli. Participants, categorized as non-alexithymic (NA), CA, or AA based on the Toronto Alexithymia Scale and Bermond-Vorst Alexithymia Questionnaire, completed assessments on empathy, social phobia, depression, and anxiety. They viewed images from the International Affective Picture System (IAPS), varying in emotional (unpleasant, neutral, and pleasant) and social (with humans, without humans) content, and evaluated them at the end of the experiment. Heart rate variability (HRV) and facial infrared thermal imaging (fITI) were recorded during image presentation. Psychometric analysis revealed higher social phobia avoidance and depression scores in CA compared to AA, and higher trait anxiety in CA compared to both AA and NA. CA and NA participants scored higher on empathic personal distress than AA. At the autonomic level, NA participants exhibited increased HF-HRV and decreased LF-HRV in response to emotional social stimuli, whereas CA and AA groups displayed no HRV modulation. Thermal responses revealed decreased nose temperature for non-social stimuli in CA, while AA showed the opposite pattern. HRV and fITI measures highlight distinct autonomic reactivity to socio-emotional stimulation and thus effectively differentiate cognitive and affective alexithymia. These physiological findings reinforce the psychometric distinction between CA and AA, suggesting tailored interventions for alexithymic disorders.

## Introduction

Alexithymia is defined as the difficulty in identifying, analyzing, and expressing emotional experiences [1–3]. Often associated with various psychiatric conditions such as anxiety or depression [4], alexithymia is also considered a stable personality trait that can occur independently of any clinical condition [5–7]. While the multidimensional nature of alexithymia remains debated [8,9], Morie et al., 2024; 10, 11 Preece et al., 2018, 2020], a prominent theoretical framework distinguishes two main facets of alexithymia: a so-called cognitive dimension, characterized by the inability to identify, analyze and verbalize feelings []; and an affective dimension, which involves emotional processing and the imaginative capacity to attribute emotional significance to events. Both dimensions can be measured using the Bermond-Vorst Alexithymia Questionnaire (BVAQ; [7]. The cognitive dimension can also be captured by the Toronto Alexithymia Scale [1,13], which focuses on difficulty identifying feelings, difficulty describing feelings, and externally oriented thinking. Beyond these proposed dimensions, alexithymic individuals often experience difficulties managing emotions and their consequences in interpersonal and social relationships, often accompanied by reduced empathy [14–17]. To explore these challenges, the present study adopts the BVAQ’s cognitive/affective distinction as a working framework, while acknowledging its ongoing refinement in its conceptualization.

To explain these difficulties, some authors proposed that they could be linked to alterations in the autonomic nervous system (ANS), an important brain-body interface for interoceptive and exteroceptive exchanges, also recognized to have a central role in emotional coding of social expressions [5,8]. Consequently, several studies have attempted to identify autonomic variations related to alexithymia dimensions, such as electrodermal activity, commonly indexed by skin conductance responses (SCRs; [16], and heart rate HR; [19]. In this context, several studies have used social stress tests, such as public speaking paradigms, to assess phasic autonomic reactivity in individuals with high and low levels of alexithymia (20–22], comparing alexithymic participants to non‑alexithymic controls. The results suggest lower electrodermal reactivity in the alexithymic group compared to the control group during the preparation period for speech [22], the oral presentation [21], or to picture series with human social content [16]. In addition, most studies measuring HR have not found differences between alexithymic and control groups when faced with social content or situation [20–22]. The effect of social content on autonomic activity thus appears more consistently in electrodermal than in HR measures. This discrepancy may reflect the composite regulation of HR by both sympathetic and parasympathetic branches of the ANS, in contrast to electrodermal activity, which is under exclusive sympathetic control [20,22]. Hence, some authors, based on resting-state heart rate variability (HRV), have suggested that alexithymic individuals may have specific deficits in parasympathetic control [23,24]. In this context, HRV is widely used as a non-invasive index of parasympathetically induced changes in consecutive heartbeats [23]. In practice, vagally mediated HRV is often indexed by time‑domain measures such as the root mean square of successive differences (RMSSD) or by high‑frequency (HF) power, whereas low‑frequency (LF) power reflects a mixture of sympathetic and parasympathetic influences. The commonly used LF/HF ratio has been criticized for oversimplifying autonomic regulation and for its context‑dependence [25,26]; therefore, in the present study, we focus on HR, RMSSD, HF‑HRV and LF‑HRV to characterize autonomic dynamics. Individuals with high resting HRV have been described as more efficient at regulating emotional and cognitive processes [27,28] and more efficient at regulating cognitive processes necessary for interpreting the stimulation [23]. In this sense, higher alexithymia scores have been associated with attenuated HF-HRV and increased LF-HRV in alexithymic individuals during an anger recall task, interpreted as attenuated sympathetic activation and diminished vagal withdrawal [29], although the precise autonomic meaning of LF/HF remains debated. However, these measures have not yet been assessed using social and non-social stimulation in alexithymia dimensions, and the analysis of indices of both sympathetic and parasympathetic activities would be beneficial in understanding the difficulties that individuals with alexithymia experience in a social context.

More recently, facial thermal variations measured by functional infrared thermal imaging (fITI), have emerged as a promising index of sympathetic activity. Unlike electrodermal activity, which reflects eccrine sweat gland activity in the palms, fITI captures sympathetically mediated vasoconstriction at the nose tip, producing temperature decreases that correlate with the arousal dimension of emotional stimuli [30,31], but not with the valence dimension [32]. These thermal variations show a convergent pattern with electrodermal activity, a classic sympathetic index, confirming their validity as markers of arousal-related reactivity [30]. Moreover, fITI offers practical advantages over traditional electrodermal measures: it is entirely contactless, minimizes participant reactivity to instrumentation, and shows slower habituation during prolonged stimulation [30,33].

Given the central role of facial expressions in social communication, fITI is particularly well-suited to investigate autonomic responses to social stimuli. Indeed, fITI has been used in social contexts, where thermal patterns vary as a function of interpersonal engagement [34,35]. For example, in women with breast cancer, higher alexithymia scores have been linked to distinct facial temperature changes during emotional picture viewing [31]. These findings suggest that skin temperature changes could be a relevant index of sympathetic activity in response to social stimuli. However, no study has yet examined how cognitive and affective alexithymia dimensions modulate facial thermal responses to social versus non‑social emotional stimuli.

Dimensional models distinguish cognitive alexithymia (CA; difficulties identifying, analyzing, and verbalizing emotions) from affective alexithymia (AA; reduced fantasizing and emotionalizing), which show partially distinct neural (*e.g.*, regional brain volumes) and behavioral correlates (*e.g.*, accuracy and reaction times in emotion‑recognition tasks) [5,15,36],). CA is more consistently associated with impaired processing of emotional and social cues, whereas AA shows less consistent associations with autonomic indices. In line with this, Martínez‑Velázquez et al. [16] reported that individuals with CA show reduced electrodermal reactivity to social stimuli compared to non‑social stimuli, whereas non‑alexithymic (NA) individuals show the opposite pattern, and AA individuals display a blunted social modulation. These data suggest that the cognitive component of alexithymia is particularly associated with an attenuated autonomic sensitivity to the social relevance of emotional stimuli.

Considering the above, the present study aims to explore autonomic emotional reactivity to social and non-social stimuli, in individuals with predominant cognitive or affective alexithymia, as well as non-alexithymic control participants. Two complementary autonomic indices were used: HRV, as an index of sympathovagal influence on HR, and fITI, as an index of sympathetic‑driven changes in cutaneous blood flow. We focused on phasic changes in HRV elicited by picture viewing, rather than resting‑state HRV, to capture task‑related adjustments in parasympathetic regulation to social versus non‑social emotional content. Recording both variables offers a dual advantage: it focuses on facial autonomic reactivity, a crucial interface for human social exchanges; and it allows us to explore the interplay between sympathetic and parasympathetic responses, in relation to alexithymia dimensions.

Building on this literature, we derived the following hypotheses:

**H1 (HRV: group × content).** Building on evidence that lower vagally mediated HRV and reduced vagal flexibility are linked to higher alexithymia and poorer emotion regulation, and that CA is particularly associated with impaired processing of social-emotional cues, we expect a significant interaction between Group (NA, AA, CA) and Content (social vs non‑social) on HRV. Specifically, CA individuals are expected to show reduced HRV modulation by social versus non‑social stimuli compared with non‑alexithymic controls, indicating less adaptive parasympathetic adjustment in socially relevant contexts.

**H2 (HRV: AA versus NA).** Given the less consistent evidence for autonomic dysregulation in AA, we tentatively expect AA participants to show HRV modulation by social versus non-social content that is closer to non-alexithymic controls than to CA individuals. This hypothesis is exploratory, as direct HRV comparisons between AA and CA are lacking.

**H3 (fITI: group × content).** For sympathetic responses indexed by fITI, we expect a Group × Content interaction. Extending findings of reduced electrodermal responses to social stimuli in CA, we hypothesize that CA individuals will show attenuated facial temperature changes to social relative to non‑social images, reflecting reduced sympathetic engagement with socially salient stimuli.

**H4 (fITI – AA versus NA).** In line with proposals that AA involves a different and possibly less pronounced autonomic profile than CA, we expect AA participants to exhibit facial thermal responses more similar to non‑alexithymic controls than to CA individuals. Given the scarcity of thermographic data in alexithymia, this hypothesis is also considered exploratory.

Together, these hypotheses test whether cognitive and affective alexithymia are associated with distinct parasympathetic (HRV) and sympathetic (fITI) response profiles to social versus non‑social emotional stimuli, and whether HRV and facial thermography provide complementary physiological markers of these dimensions.

## Materials and methods

The study was approved by the Ethics Committee of the University of Lille [Decision n° 215–6-S29], all participants gave written informed consent in accordance with the Declaration of Helsinki and received a compensation of 20€.

### Participants

Eighty healthy and unmedicated participants were recruited for the study through an online questionnaire. All participants were French speakers with normal or corrected-to-normal vision [37]. None of the participants reported any psychiatric or neurological disorders. Participants were divided into three groups: without alexithymia (NA), with affective alexithymia (AA), and with cognitive alexithymia (CA), based on their scores on the TAS-20 and the affective (A-BVAQ) and cognitive (C-BVAQ) subscales of the BVAQ [38–40]. The inclusion criteria were as follows: For the group without alexithymia (NA): TAS ≤ 44, A-BVAQ ≤ 44, and C-BVAQ ≤ 64; for the group with affective alexithymia (AA): A-BVAQ > 44 and C-BVAQ ≤ 64; and for the group with cognitive alexithymia (CA): A-BVAQ ≤ 44 and C-BVAQ > 64 [16].

The TAS-20 [38] is a self-questionnaire that comprises 20 items distributed in three sub-scales: difficulty in identifying feelings, difficulty in describing feelings, and the presence of externally oriented thinking. The participants completed each item on a 5-point scale from ‘strongly disagree’ to ‘strongly agree’. The BVAQ was also applied to distinguish between the affective and the cognitive dimensions of alexithymia [7]. The BVAQ is a self-report questionnaire that includes 40 items in five sub-scales, three related to the cognitive dimension (C-BVAQ: “Verbalizing,” “Identifying,” and “Analyzing emotions”) and two related to an affective dimension (A-BVAQ: “Emotionalizing” and “Fantasizing”). Both TAS-20 and BVAQ have been validated in the French population [41,42] and both proved to have high validity in assessing alexithymia [12]. The validity of the two-factor structure of BVAQ has been confirmed through factor analyses [39,43] but see Bagby et al., [44] for failure to support the two-factor structure).

Participants who met these criteria were invited to participate in the experiment between 9:00 AM and 12:00 PM at the IrDive Platform Imaginarium (University of Lille). Of the 80 recruited participants, 35 were excluded to ensure complete, high-quality datasets across all measures. Exclusion criteria included: excessive artifacts in physiological recordings, following established guidelines for HRV research [45,46] (thermal/ECG; n = 13), incomplete psychometric assessments (n = 6), missing subjective stimulus ratings (n = 5), technical malfunctions during presentation (n = 5), and randomization sequence errors (n = 6). The final sample consisted of 45 healthy participants aged between 19 and 34 years (M = 22.5, SD = 3; 21 females and 24 males). There were no significant differences in demographic data regarding mean age, F(2, 42) = 1.28, p = .290, η²p = .057, with mean ages of 23 for NA, 21 for CA, and 23 for AA groups. Similarly, there were no significant differences in sex ratio, χ²(2) = 2.55, p = .279, with the NA group consisting of 6 women and 10 men, the CA group of 6 women and 9 men, and the AA group of 9 women and 5 men. Sample size adequacy is evaluated through sensitivity analysis in the Statistical Analysis section.

### Empathy and clinical questionnaires

Empathy, as well as some clinical (social phobia, anxiety, and depression) characteristics of participants, were evaluated to study their relationship with alexithymia [4]. These characteristics were measured by self-administered questionnaires. The components of empathy were evaluated through the Interpersonal Reactivity Index (IRI; Davis, [47], a widely used multidimensional measure of empathy comprising four subscales:: Perspective Taking (PT), Fantasy (FS), Personal Distress (PD), and Empathic Concern (EC). The severity of social anxiety was assessed using the Social Phobia Scale [48], which consists of two subscales: Anxiety and Avoidance. The Anxiety subscale measures the level of fear or nervousness experienced in social situations, while the Avoidance subscale evaluates the extent to which individuals avoid these situations. To assess anxiety and depression, we used the State-Trait Anxiety Inventory (STAI), which includes the STAI-A for state anxiety and the STAI-B for trait anxiety [49], as well as the Beck Depression Inventory-II (BDI-II) for depressive symptoms [50]. The internal consistency (Cronbach’s α) of all scales and subscales was assessed on the final sample (N = 45) to verify their reliability in our study population (see Results section).

### Stimuli

We selected 120 pictures (1024 X 768 pixels) from the International Affective Picture System (IAPS; [51], distributed in two blocks differing by their social relevance, *i.e.*, the social content of the pictures (SCP). In line with previous studies on social perception using the IAPS pictures [16,52,53], we operationalized “social relevance” as the visible presence of human conspecifics. Accordingly, the social block (SO) included pictures depicting one or more human beings, while the non-social block (NS) comprised pictures of animals or inanimate objects. We aimed to contrast conspecific, person-related content with non‑human content. Each block included 60 pictures, distributed into three sets (3 sets X 20 pictures) differing by the emotional content of the pictures (ECP): unpleasant (U), neutral (N), and pleasant (P). To validate our stimulus selection, we analyzed the normative arousal ratings provided in the IAPS database [51]. As expected, emotional pictures (U and P combined) had arousal ratings 63% higher than neutral pictures (N), irrespective of social content. Specifically, emotional pictures (U and P combined) had arousal ratings 63% higher than neutral pictures (N), regardless of social content. This arousal difference was statistically significant, F(1, 114) = 203.99, p < .001, ηp² = .64, and did not vary between social and non-social blocks, (Social Content × Emotional Content interaction: F(1, 114) = 0.24, p = .623, ηp² < .01). These results confirmed that our picture selection successfully operationalized a pure arousal dimension (high vs. low arousal) independent of valence and social context, providing evidence that emotional responses to stimuli were comparable across social categories, and justifying the planned contrast approach described in the Statistical Analysis section.

### Recordings

The electrocardiogram (ECG) was recorded using a BIOPAC MP150 system with BIOPAC AcKnowledge 4.1 software at a sampling rate of 500 Hz and a DI modified bypass, with the Ag/AgCl pre-gelled surface electrodes (BIOPAC EL503, 7% NaCl) placed on the participant’s left and right wrists. A band-pass filter was set between 0.5 and 66.5 Hz.

An infrared thermal imaging camera, FLIR SC5000 (FLIR Systems, USA), along with Research IR 4.0 software, was used to record skin temperature. The spatial resolution of the camera was 320 × 256 ppi, and the temperature resolution was 0.01°C, with emissivity set at 0.98 [54]. Thermal video recordings were sampled at 25 Hz. A 3 x 3 pixels square was used as a region of interest (ROI), which was placed offline on the video at the tip of the nose due to its association with vasomotor sympathetic activity mediated by noradrenergic fibers. Activation of these fibers leads to vasoconstriction and a subsequent decrease in local temperature [30]. Temperature data were extracted from this ROI as a function of time and down-sampled to 5 Hz for processing.

### Procedure and task

The experimental procedure was divided into three steps. The first step was dedicated to the acclimatization of the participants to the environment. The room temperature in the laboratory was constantly held at 24°C throughout the study, and a 20-minute acclimatization period was planned before the experiment. During this time, the participants completed interviews and filled out the state anxiety, depression, and empathy questionnaires. The task was then explained orally to the participants. The second step concerned the recordings during the stimulation procedure. Participants were seated at a distance of 77 cm from the screen, similar to previous studies [30,55]. The presentation of the pictures was organized into two blocks separated by a 5-minute break: one SO block, and one NS block.

**Fig 1:**
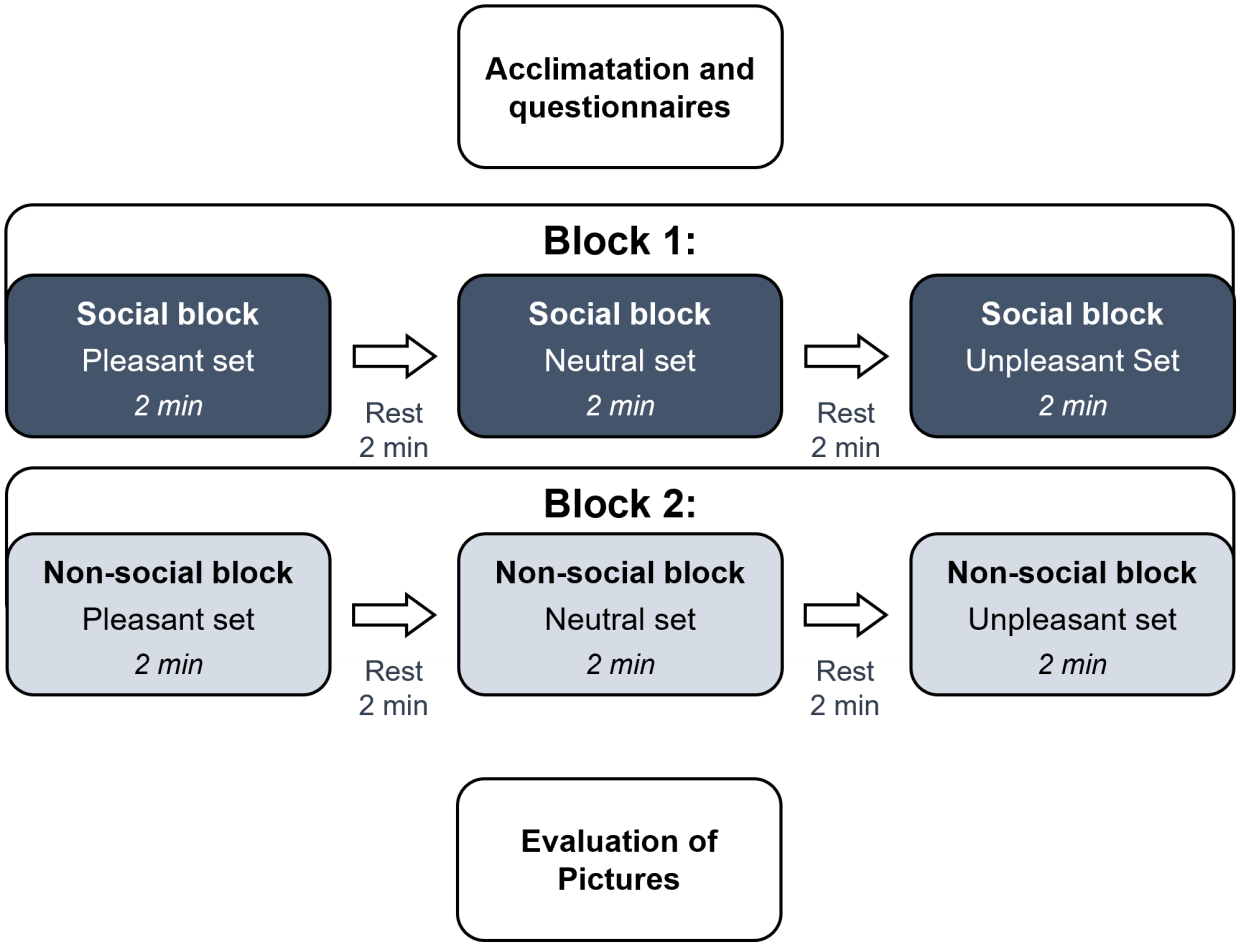
Experimental Design. The procedure was divided into three main steps: acclimation and questionnaires, two experimental blocks, and the evaluation phase. Initially, participants acclimated to the environment and completed questionnaires. Following this, they underwent two blocks of pictures viewing sessions, each consisting of three sets of images (pleasant, neutral, and unpleasant), each set consisting of a consecutive presentation of 20 pictures lasting for 2 minutes and followed by a 2-minute resting period. Block 1 presented images with social content, while Block 2 presented non-social images. The order of block presentations and emotional conditions was counterbalanced across the participants. Finally, participants evaluated the valence and arousal of all pictures using two 9-point scales.

**Fig 2:**
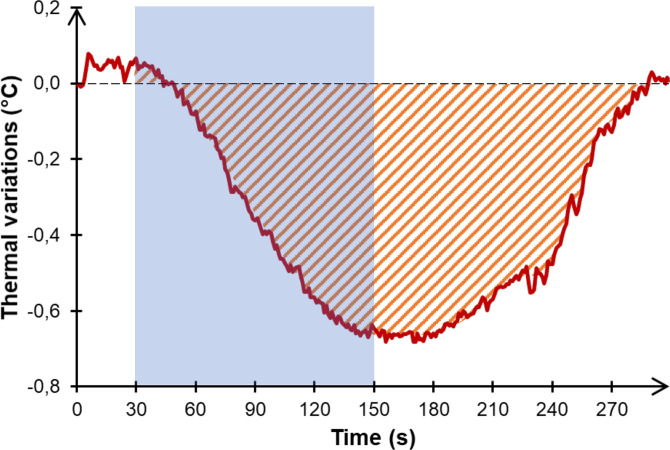
Example of a thermal response. Typical thermal variations as a function of time (red line) for one NA participant in response to the unpleasant set of the social block. The blue shaded part represents the 2-minute stimulation period. The hatched area represents the integral of temperature variations.

Each block included the presentation of U, N, and P sets (see Fig 1), each set included the presentation of 20 pictures of the same valence. Each picture was projected for 4 s and followed by: a central asterisk for 500 ms; a black screen for 500 ms; an arrow, oriented to the left (<) or the right (>), for 500 ms; and finally, a black screen for 500 ms. Participants were instructed to remain as still as possible and to respond by clicking a computer mouse according to the direction of the arrow to maintain their gaze and attention on the screen (behavioral responses were not recorded). Each set lasted 2 minutes and was followed by a 2-minute resting period of a black screen to allow the thermal response to build up. Cardiac and thermal responses were recorded during the whole duration of both blocks and rest periods. Physiological data were analyzed over these 4-minute windows (2-min stimulation + 2-min recovery) for both thermal and cardiac signals. This approach was motivated by the slow build-up and recovery dynamics of facial thermal responses [30]; see Fig 2) and ensured consistency with established short-term HRV analysis standards [45,46].

At the end of the task, and in order to control the picture selection, participants were asked to view all pictures again and to rate their valence and arousal values using two 9-point SAM scales, each varying from 1 to 9 (Self-Assessment Manikin; [56].

The order of the two blocks and emotional conditions was counterbalanced across the participants. The percentages of participants assigned to the six different emotional sets orders (NUP, PNU, PUN, UNP, UPN, NPU) did not differ significantly within each group: NA, χ²(5) = 0.81, p = .976; CA, χ²(5) = 0.00, p = 1.00; AA, χ²(5) = 6.07, p = .299. Additionally, the distributions of the emotional sets’ orders did not differ significantly between the NA and AA groups, χ²(5) = 2.94, p = 0.709, the NA and CA groups, χ²(5) = 0.67, p = .985, or the AA and CA groups, χ²(5) = 2.03, p = 0.845.

Environmental controls included: (1) room temperature maintained at 24°C (thermoneutral zone; consistent with Kosonogov et al., [30]; Nhan and Chau, [57], (2) ventilation system deactivated during recordings to eliminate thermal convection, and (3) ambient temperature continuously monitored via a region of interest on the wall captured by the thermal camera. Systematic inspection confirmed temperature stability across all sessions.

### Data analysis

#### HRV data.

We analyzed the heart rate variability (HRV) during the 4-minute periods, comprising 2 minutes of stimulus and 2 minutes of post-stimulus periods. Semi-automatic detection of R-R intervals and HRV quantification were performed using Kubios HRV 3.0.2 software. A piecewise cubic spline interpolation, set at 4 Hz, was applied to create an equidistantly sampled time series from the non-equidistantly sampled R-R interval data. The R-R intervals were detrended using a smoothness-prior method to remove the slow (< 0.04 Hz) non-stationary trends from the HRV signal. Detected ectopic beats were corrected by replacing the corrupted R-R intervals with interpolated values. For the time domain analysis, we computed the mean heart rate (HR), the maximum and the minimum HR, the standard deviation of HR, and the root mean square of successive R-R interval differences (RMSSD). For the frequency domain analysis, a power spectrum density analysis was conducted on the R-R interval series using the Fast Fourier Transform (FFT) method. The low frequency (LF) band was set at 0.04–0.15 Hz, and the high frequency (HF) band was set at 0.15–0.4 Hz. Relative powers of LF and HF bands were calculated for statistical analysis as follows: LF [%] = LF [ms²]/ total power [ms²] x 100%; HF [%] = HF [ms²]/ total power [ms²] x 100%.

#### Thermal data.

Involuntary head movements by participants occasionally introduced artifacts in the fixed Region of Interest (ROI). These artifacts were identified and corrected using either linear or polynomial interpolation, chosen based on the signal’s trend in the surrounding data. Of the 45 participants, 17 required corrections for such artifacts (8 NA, 5 AA, and 4 CA). For each participant, thermal variations in response to each set were analyzed by first subtracting the average nasal temperature recorded during a 30-second pre-set period from the subsequent 4-minute data period (Fig 2). Following this baseline correction, the integral of thermal variation was computed using the following formula, where *f (t)* represents the thermal variation waveform over time:


$$\text{Integral }{(}^{\circ }\text{C}\cdot \text{s})=\;\sum_{\text{i}=\text{1}}^{\text{n}-\text{1}}\left[\text{f}\left({\text{t}}_{\text{i}}\right)+\text{f}\left({\text{t}}_{\text{i }+\;\text{1}}\right)\right]\times \;\frac{{\Delta \text{t}}_{\text{i}}}{\text{2}}.$$

### Statistical analysis

Age and psychometric scores (TAS-20, A-BVAQ, C-BVAQ, BDI-II, STAI-A, STAI-B, IRI sub-scales) were analyzed by comparing group means (NA, AA, CA) using one-way ANOVA, followed by Bonferroni post hoc tests for pairwise comparisons. Subjective arousal, integral of thermal variations, and HRV parameters were analyzed using a three-way repeated-measures design, considering the factors ECP (U, N, P), SCP (SO, NS), and group (NA, AA, CA). Our hypotheses centered on emotional arousal as the key modulator of autonomic responses (H2-H3), consistent with dimensional models distinguishing arousal from valence [32,58]. We therefore specified planned orthogonal contrasts: (1) for the Social Content factor (2 levels), a linear contrast [Social(+1), Non-Social(−1)]] tested the main effect of social content; (2) for the Emotional Content factor (3 levels), a quadratic contrast [(Unpleasant + Pleasant)/2 − Neutral] isolated emotional arousal effects,

independent of valence direction (see D’Hondt et al., [59]. This quadratic specification aligns with established practices in emotion research [58], evidence that peripheral autonomic measures primarily track arousal intensity rather than valence [60,61] and matched our stimulus selection where unpleasant and pleasant pictures were equivalently arousing (IAPS ratings 63% higher than neutral). By directly targeting the specific hypothesis on emotional arousal effects (high vs. low arousal), this approach enables more precise and powerful statistical tests than global analyses that treat all emotional conditions as equivalent [62,63]. The three-way interaction (Group × Social Content Linear × Arousal Quadratic) constituted our primary hypothesis test. To evaluate the adequacy of our sample size for detecting the hypothesized three-way interaction, we conducted a sensitivity analysis using G*Power software [64]. For the omnibus Group × Social Content × Emotional Content interaction in our mixed-design ANOVA (3 between-subjects groups × 6 within-subjects conditions), we specified the following parameters: α = .05, power (1 – β) =.80, total N = 45, number of groups = 3, number of repeated measurements = 6, and assumed correlation among repeated measures r = .50 (a typical value for psychophysiological data; [65].The analysis indicated that the study could detect an interaction effect size of f = 0.177 (equivalent to ηp² = .030), corresponding to a small-to-medium effect according to Cohen’s [66] benchmarks (critical F(10, 210) = 1.88). This threshold reflects the minimum effect size detectable with 80% power under the specified design. Correlations between variables were tested using Pearson’s coefficient (r) for the entire sample and Spearman’s coefficient (ρ) within groups with fewer than 30 participants. Statistical significance was set at p ≤ .05.

## Results

### Psychometric properties

#### Internal consistency.

All questionnaires demonstrated acceptable to excellent internal consistency in our sample (N = 45). For alexithymia measures, the TAS-20 showed good reliability (α = .84), as did the BVAQ-Affective dimension (α = .82) and BVAQ-Cognitive dimension (α = .89). Empathy subscales (IRI) yielded the following coefficients: Fantasy Scale (α = .76), Personal Distress (α = .71), Perspective Taking (α = .64), and Empathic Concern (α = .75). Clinical measures all demonstrated good to excellent reliability: Social Phobia Anxiety subscale (α = .89), Social Phobia Avoidance subscale (α = .91), STAI-A (α = .89), STAI-B (α = .92), and BDI-II (α = .88). These coefficients are consistent with previous validation studies and confirm the psychometric adequacy of the instruments for our research questions.

#### Alexithymia scores.

As expected, TAS-20 and C-BVAQ scores were strongly correlated, r(47) = 0.88, p < .001, supporting the convergent validity of cognitive alexithymia measures. In contrast, A-BVAQ scores did not correlate with either the TAS-20 or C-BVAQ scores (ps > .450), confirming the distinctiveness of affective alexithymia as assessed by the BVAQ.

#### Empathy and clinical measures.

Regarding the empathy scale (IRI), an effect of the group was observed on the PD, *F*(2,42) = 11.94, p < .001, η²p = .36, and FS subscales *F*(2,42) = 4.82, p = .013, η²p = .19. Participants in the AA group showed lower PD scores than NA participants (p = .017) and CA participants (p < .001), and lower FS scores than NA participants (p = .023) and CA participants (p = .037). There were no significant differences between the groups on the PT (*F* < 1.00, p = .750), and EC (*F* = 1.10, p = .344) subscales.

The avoidance subscale of the Social Phobia Scale differed significantly across groups (*F*(2, 42) = 4.83, p = .013, η²p = .19). Specifically, the CA group exhibited higher scores compared to the AA group (p = .012). No significant group differences were found on the STAI-A scale (*F*(2, 42) = 2.60, p = .086, η²p = .11). However, on the STAI-B scale (*F*(2, 42) = 8.15, p = .001, η²p = .28), the CA group scored higher than both the NA (p = .018) and AA groups (p = .001). There were no significant differences between the NA and AA groups on these scales (p > .13). Regarding depression scores (BDI, *F*(2, 42) = 6.08, p = .005, η²p = .23), the CA group also scored higher than the AA group (p = .004), but not significantly higher than the NA group (p = .11). The AA and NA groups did not differ significantly on the depression scale (p = .54). Table 1 summarizes empathy and clinical measures.

**Table 1:** Empathy and clinical scores of the three groups: without alexithymia (NA), and with affective (AA) and cognitive alexithymia (CA). IRI = Interpersonal Reactivity Index, IRI sub-scales: IRI-FS = Fantasy, IRI-PD = Personal Distress, IRI-PT = Perspective Taking, and IRI-EC = Empathic Concern; SP = Social Phobia; STAI = State-Trait Anxiety Inventory; BDI-II = Beck Depression Inventory-II, SD = standard deviation. Values in the same row not sharing the same index differ significantly at p < .05 in the bilateral equality test for column means. The tests are adjusted for all pairwise comparisons within a row using Bonferroni correction.

	NA N = 16 mean (SD)	CA N = 15 mean (SD)	AA N = 14 mean (SD)
**IRI-FS**	34.3_b_ (3.3)	33.9_a,b_ (6.8)	26.7_a_ (10.7)
**IRI-PD**	24.2_b_ (6.8)	29.3_b_ (7.2)	17.0_a_ (6.3)
**IRI-PT**	36.4_a_ (7)	35.1_a_ (7.5)	34.1_a_ (10.3)
**IRI-EC**	37.3_a_ (6.1)	34.4_a_ (8.1)	32.6_a_ (11.6)
**SP-ANXIETY**	19.1_a_ (9.5)	26.4_a_ (9.8)	19.1_a_ (10.4)
**SP-AVOIDANCE**	16.4_a,b_ (10.6)	24.9_b_ (11.1)	12.1_a_ (12.1)
**STAI-A**	30,7_a_ (7.8)	35.7_a_ (8.9)	30.1_a_ (4.8)
**STAI-B**	37.9_a_ (9.2)	47.5_b_ (12)	34.2_a_ (4.6)
**BDI-II**	7.6_a,b_ (6.7)	12.5_b_ (7.8)	4.5_a_ (3.1)

#### Subjective arousing value of pictures.

Group differences in mean arousal judgments of the pictures were non-significant (*F*(2, 42) = 0.17, p = .846, η²p = .008). Additionally, the emotional content of pictures (ECP) did not differ on arousal judgments across all three groups (*F*(4, 84) = 1.17, p = .328, η²p = .053). As anticipated, arousal ratings were significantly higher (by 59%) for emotional pictures compared to neutral ones. This arousal contrast (quadratic contrast, *F*(1, 42) = 151.21, p < .001, η²p = .784) accounted for 77% of the variance associated with the ECP factor.

The social context of pictures (SCP) also influenced arousal judgments (*F*(1, 42) = 11.43, p = .002, η²p = .214) similarly across all three groups (*F*(2, 84) = 2.13, p = .132, η²p = .092). There was no significant interaction effect between ECP and SCP factors (*F*(2, 84) = 2.01, p = .140, η²p = .046) across groups (*F*(4, 84) = 1.55, p = .195, η²p = .069).

#### Autonomic reactivity to socio-emotional stimulation.

We tested our four hypotheses using planned orthogonal contrasts on HRV and fITI measures. We first assessed whether the modulation of responses by emotional arousal differed between social and non-social contexts across the three groups (H1 for HRV; H3 for fITI), then specifically compared the affective alexithymia (AA) group to non-alexithymic (NA) controls (H2 for HRV; H4 for fITI).

#### Differential HRV responsivity to social content.

**Fig 3:**
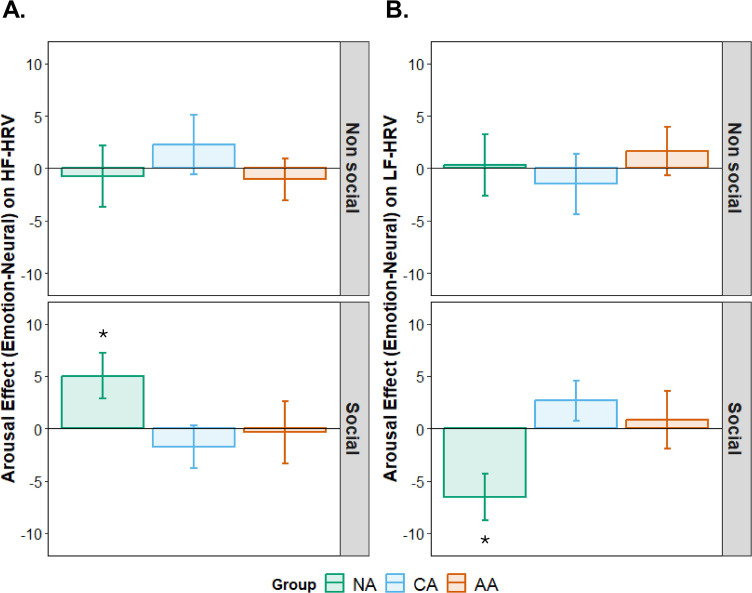
Interaction Effects of Social and Emotional Context on LF-HRV and HF-HRV in non-alexithymia (NA), cognitive (CA) and affective (AA) alexithymia. The arousal effect (Emotion – Neutral) on the **A)** high frequencies (HF-HRV) and **B)** low frequencies (LF-HRV) heart rate variability in NA (green), CA (blue) and AA (orange) for non-social (top panels) and social sets (bottom panels). Positive values indicate greater HF-HRV or LF-HRV for emotional compared to neutral sets. Error bars represent the standard error of the mean.

The three-way interaction contrast (Group × Social Content Linear × Arousal Quadratic) was significant for both HF-HRV, *F*(2, 42) = 3.53, *p* = .038, η²p = .144 (Fig 3A), and LF-HRV, *F*(2, 42) = 3.52, *p* = .039, η²p = .143 (Fig 3B). We decomposed this interaction by examining whether the effect of social content on arousal reactivity differed across groups.

**Non-Alexithymia (NA).** The Social Content Linear × Arousal Quadratic interaction was significant for both HF-HRV, *F*(1, 15) = 5.40, *p* = .035, η²p = .265, and LF-HRV, *F*(1, 15) = 5.34, *p* = .035, η²p = .263. In the social condition, emotional pictures elicited higher HF-HRV (M = 45.2 n.u., SD = 18.3) compared to neutral pictures (M = 38.7 n.u., SD = 16.9), *F*(1, 15) = 6.22, p = .025, η²p = .293, and lower LF-HRV (emotional: M = 54.8 n.u., SD = 18.3; neutral: M = 61.3 n.u., SD = 16.9), *F*(1, 15) = 6.19, p = .025, η²p = .292. In the non-social condition, arousal effects were non-significant (HF-HRV: *F*(1, 15) = 0.06, p = .818; LF-HRV: *F*(1, 15) = 0.05, p = .822).

**Cognitive Alexithymia (CA).** The Social Content Linear × Arousal Quadratic interaction was non-significant (HF-HRV: *F*(1, 14) = 2.27, p = .155, η²p = .140; LF-HRV: *F*(1, 14) = 2.27, p = .154, η²p = .139). Arousal effects were non-significant in both social (HF-HRV: *F*(1, 14) = 1.15, p = .301; LF-HRV: *F*(1, 14) = 1.15, p = .302) and non-social conditions (HF-HRV: *F*(1, 14) = 0.50, p = .491; LF-HRV: *F*(1, 14) = 0.51, p = .489).

**Affective Alexithymia (AA).** The Social Content Linear × Arousal Quadratic interaction was non-significant (HF-HRV: *F*(1, 13) = 0.12, *p* = .731, η²p = .009; LF-HRV: *F*(1, 13) = 0.10, *p* = .758, η²p = .008). Arousal effects were non-significant in both social (HF-HRV: *F*(1, 13) = 0.04, *p* = .837; LF-HRV: *F*(1, 13) = 0.06, *p* = .810) and non-social contexts (HF-HRV: *F*(1, 13) = 0.59, *p* = .456; LF-HRV: *F*(1, 13) = 0.58, *p* = .461).

To summarize, the NA group exhibited significant modulation of HRV by emotional arousal exclusively during social picture viewing. In contrast, both CA and AA groups showed no significant arousal effects in either condition. HF-HRV reflects parasympathetic cardiac control; its increase alongside the LF-HRV decrease in NA during social-emotional viewing indicates enhanced vagal modulation in this context.

#### Differential thermal responsivity to social content.

**Fig 4:**
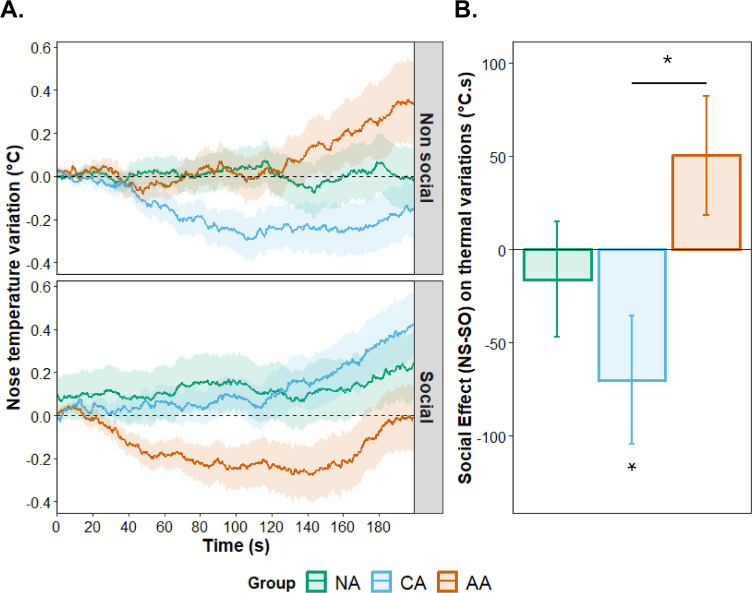
Effect of the social content on thermal variations in non-alexithymia, cognitive and affective alexithymia. **A)** Thermal variations as a function of time in NA (green), CA (blue), and AA (orange) for non-social (top panel) and social sets (bottom panel). **B)** The social content effect (NS – SO) on the integral of the thermal variations in NA (green), CA (blue), and AA (orange). For panel B), Positive values indicate greater thermal variations for non-social compared to social sets. In this context, negative values indicate a greater decrease in nose temperature in response to non-social stimulation compared to social stimulation. Error bars represent the standard error of the mean.

The three-way interaction contrast (Group × Social Content Linear × Arousal Quadratic) was not significant, *F*(4, 82) = 0.63, *p* = .642, η²p = .030. We therefore tested the two-way Group × Social Content interaction, collapsing across emotional arousal levels. This interaction was significant, *F*(2, 41) = 3.42, *p* = .042, η²p = .143 (Fig 4). We decomposed this interaction by examining the social content effect within each group.

**Non-Alexithymia (NA).** The social content effect was not significant, *F*(1, 14) = 0.03, *p* = .869, η²p = .002 (*M*social = −85.2 °C·s, *SD* = 89.4; *M*non-social = −91.8 °C·s, *SD* = 68.6).

**Cognitive Alexithymia (CA).** Nasal temperature was lower in the non-social condition (*M* = −123.4 °C·s, *SD* = 98.6) compared to the social condition (*M* = −18.7 °C·s, *SD* = 102.3), *F*(1, 14) = 4.89, *p* = .044, η²p = .259.

**Affective Alexithymia (AA).** Nasal temperature showed a trend in the opposite direction: lower in the social condition (*M* = −156.3 °C·s, *SD* = 115.8) compared to the non-social condition (*M* = −67.7 °C·s, *SD* = 71.9), although this difference was not significant, *F*(1, 13) = 2.94, *p* = .110, η²p = .184. A planned contrast directly comparing CA versus AA on the social content effect (SO − NS) was significant, *F*(1, 41) = 6.73, *p* = .013, η²p = .141, while the planned contrast comparing AA versus NA groups on the social content effect (SO − NS) was not significant, F(1, 41) = 2.67, p = .110, η²p = .061.

To summarize, the CA group showed greater temperature decrease (vasoconstriction) for non-social stimuli, whereas AA showed a trend toward greater decrease for social stimuli. The CA vs. AA contrast confirmed opposite thermal patterns. Nasal temperature changes reflect sympathetically mediated cutaneous blood flow; decreases indicate vasoconstriction associated with sympathetic activation.

#### Psychophysiological correlates across alexithymia dimensions.

**Fig 5:**
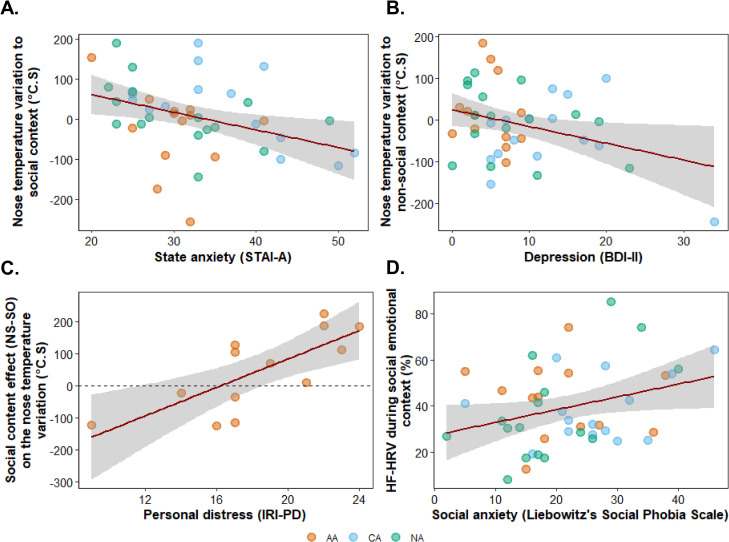
Psychophysiological Correlates Across Social and Emotional Contexts. Correlations between psychological measures and physiological responses. **(A)** Nose temperature variation in social context versus state anxiety (STAI-A). **(B)** Nose temperature variation in a non-social context versus depression (BDI-II). **(C)** Social content effect (NS – SO) on nose temperature variation versus personal distress (IRI-PD) for affective alexithymia (AA) participants. Positive values indicate greater thermal variations for non-social (NS) compared to social (SO) sets. Negative values indicate a greater decrease in nose temperature for the social compared to the non-social sets. **(D)** High-frequency heart rate variability (HF-HRV) during social emotional context versus social anxiety (Liebowitz’s Social Phobia Scale). Red lines represent linear regression fits, with gray areas indicating 95% confidence intervals. Each dot represents an individual participant’s data point. AA = participants with affective alexithymia (orange), CA = participants with cognitive alexithymia (blue), NA = participants without alexithymia (green).

The thermal responses in the social condition correlated with state-anxiety scores [STAI-A: r(43) = −0.36; p = .015, Fig 5A]. The greater the state anxiety scores were, the greater was the decrease in temperature at the tip of the nose in response to social content. This correlation was observed in NA [ρ(14) = −0.61; p = .012] and in CA [ρ (13) = −0.56; p = .031] but not in AA [ρ(12) = −0.25; p = .389]. The thermal responses in the non-social condition correlated with depression scores [BDI-II: r(42) = −0.31; p = .040, Fig 5B]. The greater the depression, the greater was the decrease in temperature in response to non-social content.

In AA, the effect of SCP on the thermal responses negatively correlated with the personal distress [ρ(11) = −0.78; p = .002, Fig 5C] scale of the IRI. As personal distress increases, the difference in thermal variations between social and non-social stimuli decreases. Positive values on the y-axis indicate greater thermal variations for social compared to non-social sets, suggesting that participants with higher personal distress scores exhibited a less pronounced physiological response (greater nose temperature variation) to social content compared to non-social content.

HF-HRV during social emotional set correlated with the anxiety subscale of the Social Phobia Scale [SP-anxiety: r(42) = 0.31; p = .042, Fig 5D]. The greater the social anxiety scores were, the greater was the HF-HRV during emotional social stimulation.

## Discussion

This study aimed to compare the effects of social and non-social visual emotional stimulation on autonomic indices in participants without alexithymia (NA), with cognitive alexithymia (CA), and with affective alexithymia (AA). In line with our general hypothesis that social stimuli would elicit distinct autonomic responses compared to non-social stimuli in alexithymia, this study allows to show a differentiated socio-emotional processing across alexithymic dimensions. In a population identified as having distinct psychometric profiles of alexithymia, two new major results can support such distinction: firstly, parasympathetic and sympathetic reactivity to social vs. non-social stimuli is different when presented to non alexithymic, cognitive or affective alexythimic participants; secondly, autonomic responses are distinctively related to empathy and clinical measures of cognitive and affective alexithymic participants when they face social and non-social contents.

### Psychometric profiles

In assessing clinical measures, distinct patterns emerged among the studied groups in terms of avoidance sub-scale of the Social Phobia scale and depression, trait-anxiety, and personal disease sub-scale of the empathy inventory. The CA group exhibited significantly higher scores in the avoidance sub-scale of social phobia and depression compared to the AA, but not significantly different from the NA group; in addition, the AA and NA groups did not differ significantly in these measures. Previous studies have also reported a positive association between symptoms of alexithymia and social phobia [67], depression [4,68,69] or both [16]. The results of the current study underscore the variability of these symptoms across affective and cognitive dimensions, but not significantly when compared to non-alexithymic individuals. Concerning anxiety measures, the CA group distinguished itself by higher trait-anxiety levels compared to both AA and NA groups, although there were no significant differences in state-anxiety levels. This aligns with previous studies that linked cognitive alexithymia assessed by TAS-20 to heightened trait anxiety [4,16,38,68]. The heightened trait anxiety and social avoidance observed in CA participants might reflect their struggles with emotional awareness and difficulties related to theory of mind impairments. Indeed, a deficit in identifying others’ emotions, linked to a deficit in theory of mind, has been observed in individuals with alexithymia [70].

Besides, CA and NA groups exhibited higher scores than the AA group on the personal distress sub-scores of the empathy inventory. This sub-scale assesses discomfort and anxiety when exposed to others’ negative emotions, indicative of emotional contagion and empathic distress [47,71]. Studies employing TAS-20 have linked higher personal distress scores with depression, suggesting a propensity towards emotional contagion in depressive states [71]. The reduced empathy PD scores observed in AA participants are in line with studies suggesting diminished empathy in affective alexithymia, possibly linked to altered socio-emotional processing mechanisms [70,72]. Taken together, obtained results lead to consider: first, further research is required to understand the lack of difference observed between NA vs. AA for trait-anxiety and vs. CA for empathic personal distress; second, and interestingly, social phobia avoidance and depression, trait anxiety and personal distress bolster arguments which take greater account of the bidimensional conception of alexithymia, cognitive and affective, as a credible basis for distinct psychometric profiles [1,16].

### Effect of social relevance on heart rate variability

Results supported the hypothesis that social emotional stimuli would elicit distinct autonomic responses compared to non-social emotional stimuli in alexithymia. Indeed, the effects of social content and emotional content of visual stimulation on HRV varied across different alexithymia groups. In the NA group, participants showed higher HF-HRV and lower LF-HRV during emotional sets compared to neutral ones in the social block, but not in the non-social block. This emotional modulation effect was not observed in the CA or AA groups, indicating differential autonomic responses to social stimuli based on alexithymia.

While differences in HR measures are not systematically found between individuals with alexithymia (assessed via TAS) and those without alexithymia [20–22], our findings are consistent with previous studies that have observed an increased HR in individuals with higher levels of alexithymia when exposed to emotionally laden stimuli such as unpleasant movies [18] or emotional provocations [24,73]. However, autonomic differences to social vs. non-social stimuli could partly reflect attentional biases rather than pure emotional reactivity. Alexithymia influences visual exploration, reducing attention to social cues in favor of non-social elements [74], and modulates insula responses to others’ pain [75]. In our study, absent HRV modulation in CA/AA during social viewing may stem from diminished engagement with social content, as subjective arousal ratings did not differ across groups. In this frame, an increase of HF-HRV, as observed in the NA group, reflects an increase of parasympathetic influence on the sinusal node and a consequent decrease in HR [76]. *A contrario*, it can be hypothesized that the absence of any increase of HF-HRV in CA and AA could reveal the difficulty of alexithymic individuals to decrease HR and, consequently, leads to a reduction in the ability to ensure emotional regulation. [24].

Indeed, parasympathetic influence on HR has been associated with emotional regulation abilities and described as a psychophysiological marker of inhibitory control [28,77,78]. From the perspective of the polyvagal theory [79], the parasympathetic influence on HR supports social communication by promoting calm and adaptive states that facilitate social interactions. In social contexts, increased HF-HRV would reflect a greater capacity for social engagement, which is crucial for effective communication and emotional connection. In this line, the NA group’s higher HF-HRV during social and emotional conditions would indicate their enhanced ability to regulate emotions and engage socially. Conversely, the absence of this effect in the CA and AA groups suggests that individuals with alexithymia, regardless of their cognitive or affective dimensions, may have impaired autonomic regulation in social contexts.

### Effect of social relevance on thermal variations

The decrease in nose temperature has been associated with autonomic adjustments linked to a sympathetic vasoconstrictive reaction, which has many adaptive functions in the context of the fight/flight response, particularly redistributing blood flow [30]. This phenomenon has been reported in response to negative emotions such as pain or fear of pain [80], negative emotional situations or distress (81), exposure to unpleasant pictures with low arousal [82] or pleasant and unpleasant with high arousal pictures [30]. Additionally, Salazar-López et al. [82] reported that nose temperature decreases in tasks involving emotional contagion, associated with an empathic response. In the present study, while no effect of the emotional context of the pictures was observed, the social context’s effect varied according to alexithymia dimensions. Participants with cognitive alexithymia showed a greater decrease in nose temperature in response to non-social compared to social stimulations, whereas participants with affective alexithymia exhibited the reverse pattern.

Regarding the absence of emotional effect on the thermal responses, in our design, unpleasant and pleasant pictures were selected to be higher in normative arousal than neutral pictures, while social and non‑social versions within each valence category were closely matched on both valence and arousal ratings. As a result, arousal mainly varied between emotional (pleasant and unpleasant) and neutral sets, whereas social content introduced an additional, orthogonal source of variance within each arousal level. Under these conditions, sympathetic thermal adjustments may have been more strongly influenced by the social relevance of the stimuli and by alexithymia dimensions than by the emotional factor itself. In other words, our findings suggest that nasal temperature in this paradigm reflects the joint influence of arousal, social context, and individual differences, rather than acting as a purely emotional‑based index of emotional intensity.

For the CA group, the sympathetic vasoconstrictive reaction to non-social content but not to social content aligns with previous findings where sympathetic reactivity (SCRs) was lower in response to social than non-social stimuli in CA individuals, with the opposite observed in Non-Alexithymia (NA) individuals [16]. These results suggest that individuals with alexithymia may use suppression, *i.e.,* modulating the emotional response which has already occurred, and inhibiting potential emotional expressions to come [83], as an emotion regulation strategy [84,85], particularly in social situations. Furthermore, during social stimulation, participants with cognitive alexithymia did not show a decrease in nasal temperature as they did in a non-social condition. Previous studies have found that variation in nose temperature occurs during social interactions, such as sexual arousal [80], interpersonal contact [34], children apologizing [81], sympathy crying [55], and viewing pleasant, low-arousal images [82]. This absence of nasal temperature variation, indicating hypoactivation of the sympathetic system [86,87], suggests that CA individuals may be inhibiting emotional physiological responses in social situations.

Conversely, AA participants exhibited a decrease in nose temperature in social contexts and an increase in non-social contexts, suggesting a hyperactivation of the sympathetic system when confronted with social situations. This pattern aligns with the negative relationship found between Personal Distress (PD) scores and the social effect on nose temperature variations in the AA group, indicating that higher PD scores are associated with greater decreases in nose temperature to social content compared to non-social content. These findings could explain the social difficulties experienced by individuals with alexithymia and have significant therapeutic implications. Thus, when we consider social situations: for AA individuals, therapeutic support could focus on training to reduce sympathetic activity and optimize emotional regulation, while for CA individuals, the emphasis could be on reducing anxiety and developing empathic skills. However, further research is needed to better disentangle empathic and clinical correlates of autonomic variations as a function of the alexithymic dimensions.

## Conclusion

This study has several methodological strengths. First, the research lies in the examination of both facets of the autonomic nervous system, parasympathetic and sympathetic, providing a comprehensive understanding of autonomic regulation in alexithymia. The use of infrared thermal imaging to measure nose temperature allowed for a precise and non-invasive assessment of sympathetic activity, adding a novel dimension to the physiological evaluation. Second, by distinguishing between cognitive and affective dimensions of alexithymia, the study offers nuanced insights into how these subtypes differentially impact physiological responses.

Despite the originality and the interest of this work, some limitations must be acknowledged. While our findings support distinct autonomic profiles for cognitive and affective alexithymia, this distinction remains theoretically debated. Recent work suggests that some components of affective alexithymia, such as low fantasy or emotional reactivity, may not be core features of the trait [10,11], and alternative models propose a tripartite structure [44] or exclude fantasy entirely (Perth Alexithymia Questionnaire; [10]. Future studies could replicate our protocol using these alternative tools to test whether the observed ANS patterns generalize beyond the BVAQ framework. Besides, the study focused on specific types of visual stimuli. Our operationalization of social content as pictures containing visible human figures, although consistent with previous work [16,52,53], does not capture all possible sources of socially interpretable content. Any contamination of the non‑social block by socially interpretable images would make the social vs. non‑social contrasts conservative; our interpretation is therefore restricted to the effect of visible conspecific presence (humans vs. non‑humans). Further research should include direct manipulation checks in which participants explicitly rate the “socialness” of the stimuli, ideally alongside physiological measures, to more directly test the relationship between perceived social relevance and emotional responding. Finally, the subjective evaluation of the arousal value of emotional and social stimuli did not depend on the groups. A possible explanation is that the emotional content of pictures was not strong enough to induce specific components of interoceptive sensibilities, necessary for differential subjective judgements in alexithymic individuals, as suggested by the studies of Ventura-Bort et al. [88] and Matsumoto et al. [89].

This study demonstrates that social emotional stimuli, when compared to non-social emotional ones, modulate autonomic responses differently depending on alexithymia dimensions. Notably, NA participants exhibited greater HF-HRV and lower LF-HRV in response to emotional social contexts, indicating enhanced parasympathetic activity. In contrast, no significant modulation of HRV was observed in the CA and AA groups in response to social stimuli, suggesting possible impairments in parasympathetic mobilization, usually required to optimize emotional behavior regulation in social contexts. Moreover, CA participants showed a greater decrease in nose temperature in response to non-social stimuli, and AA participants exhibited the opposite pattern. Thus, the combination of parasympathetic and sympathetic measures allowed to identify the differential autonomic reactivity between NA and alexithymic individuals (CA and AA) on one side and between CA and AA on the other side, respectively indiced by HRV and fITI measures. By distinguishing between cognitive and affective dimensions of alexithymia, this research offers nuanced insights into how these subtypes differentially impact physiological responses. The findings also suggest different behavioral therapeutic approaches for CA and AA individuals. For CA, interventions could focus on developing empathic skills, while for AA, the focus could be on training for emotional regulation. This underlines the importance of considering the multidimensional nature of alexithymia in both psychophysiological research and clinical practice. Finally, this study enhances our understanding of how social relevance influences autonomic reactivity across different alexithymia dimensions and strongly encourages us to further optimize autonomic distinctions to align with established psychometric classifications. This seems to be a promising way of enhancing diagnostic and remediation procedures.

## References

[1] Goerlich-Dobre KS, Bruce L, Martens S, Aleman A, Hooker CI. Distinct associations of insula and cingulate volume with the cognitive and affective dimensions of alexithymia. Neuropsychologia. 2014;53:284–92. doi: 10.1016/j.neuropsychologia.2013.12.006 24334109

[2] Sifneos PE. The prevalence of “alexithymic” characteristics in psychosomatic patients. Psychother Psychosom. 1973;22(2):255–62. doi: 10.1159/000286529 4770536

[3] van der Velde J, Servaas MN, Goerlich KS, Bruggeman R, Horton P, Costafreda SG, et al. Neural correlates of alexithymia: a meta-analysis of emotion processing studies. Neurosci Biobehav Rev. 2013;37(8):1774–85. doi: 10.1016/j.neubiorev.2013.07.008 23886515

[4] Grynberg D, Luminet O, Corneille O, Grèzes J, Berthoz S. Alexithymia in the interpersonal domain: A general deficit of empathy? Personality and Individual Differences. 2010;49(8):845–50. doi: 10.1016/j.paid.2010.07.013

[5] Bermond B, Bierman DJ, Cladder MA, Moormann PP, Vorst HCM. The cognitive and affective alexithymia dimensions in the regulation of sympathetic responses. Int J Psychophysiol. 2010;75(3):227–33. doi: 10.1016/j.ijpsycho.2009.11.004 19951721

[6] Jessimer M, Markham R. Alexithymia: a right hemisphere dysfunction specific to recognition of certain facial expressions?. Brain Cogn. 1997;34(2):246–58. doi: 10.1006/brcg.1997.0900 9220088

[7] Vorst HCM, Bermond B. Validity and reliability of the Bermond–Vorst Alexithymia Questionnaire. Personality and Individual Differences. 2001;30(3):413–34. doi: 10.1016/s0191-8869(00)00033-7

[8] Fantini-Hauwel C, Gois C, Luminet O, Banse E, Bigot A, Noel X, et al. Exploring alexithymia with the French Perth alexithymia questionnaire: latent structure, profiles, and links with affective outcomes. Front Psychol. 2025;16:1615612. doi: 10.3389/fpsyg.2025.1615612 40599529 PMC12209315

[9] Morie KP, Lord KA, Diefenbach GJ, Basuchoudhary O, Lewis S, Assaf M. Subscales of alexithymia show unique pathways through reappraisal and suppression to anxiety, depression and stress. J Affect Disord. 2024;347:445–52. doi: 10.1016/j.jad.2023.11.038 38007105 PMC10842914

[10] Preece D, Becerra R, Robinson K, Dandy J, Allan A. The psychometric assessment of alexithymia: Development and validation of the Perth Alexithymia Questionnaire. Personality and Individual Differences. 2018;132:32–44. doi: 10.1016/j.paid.2018.05.011

[11] Preece DA, Becerra R, Robinson K, Allan A, Boyes M, Chen W, et al. What is alexithymia? Using factor analysis to establish its latent structure and relationship with fantasizing and emotional reactivity. J Pers. 2020;88(6):1162–76. doi: 10.1111/jopy.12563 32463926

[12] Bermond B, Oosterveld P, Vorst HCM. Measures of Alexithymia. In: Boyle GJ, Saklofske DH, Matthews G, editors. Measures of Personality and Social Psychological Constructs. San Diego: Academic Press; 2015. p. 227–56. doi: 10.1016/B978-0-12-386915-9.00009-7

[13] Taylor GJ, Bagby RM, Parker JDA. Disorders of Affect Regulation: Alexithymia in Medical and Psychiatric Illness. Cambridge University Press; 1999.

[14] Bogdanov VB, Bogdanova OV, Gorlov DS, Gorgo YP, Dirckx JJJ, Makarchuk MY, et al. Alexithymia and empathy predict changes in autonomic arousal during affective stimulation. Cogn Behav Neurol. 2013;26(3):121–32. doi: 10.1097/WNN.0000000000000002 24077571

[15] Goerlich-Dobre KS, Lamm C, Pripfl J, Habel U, Votinov M. The left amygdala: A shared substrate of alexithymia and empathy. Neuroimage. 2015;122:20–32. doi: 10.1016/j.neuroimage.2015.08.014 26275382

[16] Martínez-Velázquez ES, Honoré J, de Zorzi L, Ramos-Loyo J, Sequeira H. Autonomic reactivity to arousing stimuli with social and non-social relevance in alexithymia. Frontiers in Psychology. 2017;8. doi: 10.3389/fpsyg.2017.00361 PMC5346581 28348539

[17] Pouga L, Berthoz S, de Gelder B, Grèzes J. Individual differences in socioaffective skills influence the neural bases of fear processing: the case of alexithymia. Hum Brain Mapp. 2010;31(10):1469–81. doi: 10.1002/hbm.20953 20127873 PMC6870595

[18] Franz M, Schaefer R, Schneider C. Psychophysiological Response Patterns of High and Low Alexithymics Under Mental and Emotional Load Conditions. Journal of Psychophysiology. 2003;17(4):203–13. doi: 10.1027/0269-8803.17.4.203

[19] Panayiotou G, Panteli M, Vlemincx E. Processing emotions in alexithymia: a systematic review of physiological markers. Alexithymia: Advances in research, theory, and clinical practice. 2018. 291–320.

[20] Hua J, Le Scanff C, Larue J, José F, Martin J-C, Devillers L, et al. Global stress response during a social stress test: impact of alexithymia and its subfactors. Psychoneuroendocrinology. 2014;50:53–61. doi: 10.1016/j.psyneuen.2014.08.003 25179321

[21] Kleiman A, Kramer KA, Wegener I, Koch AS, Geiser F, Imbierowicz K, et al. Psychophysiological decoupling in alexithymic pain disorder patients. Psychiatry Res. 2016;237:316–22. doi: 10.1016/j.psychres.2016.01.021 26804974

[22] Pollatos O, Werner NS, Duschek S, Schandry R, Matthias E, Traut-Mattausch E, et al. Differential effects of alexithymia subscales on autonomic reactivity and anxiety during social stress. J Psychosom Res. 2011;70(6):525–33. doi: 10.1016/j.jpsychores.2010.12.003 21624575

[23] Lischke A, Pahnke R, Mau-Moeller A, Behrens M, Grabe HJ, Freyberger HJ, et al. Inter-individual Differences in Heart Rate Variability Are Associated with Inter-individual Differences in Empathy and Alexithymia. Front Psychol. 2018;9:229. doi: 10.3389/fpsyg.2018.00229 29541046 PMC5836598

[24] Peasley-Miklus CE, Panayiotou G, Vrana SR. Alexithymia predicts arousal-based processing deficits and discordance between emotion response systems during emotional imagery. Emotion. 2016;16(2):164–74. doi: 10.1037/emo0000086 26461248

[25] Billman GE. The LF/HF ratio does not accurately measure cardiac sympatho-vagal balance. Front Physiol. 2013;4:26. doi: 10.3389/fphys.2013.00026 23431279 PMC3576706

[26] Reyes del Paso GA, Langewitz W, Mulder LJM, van Roon A, Duschek S. The utility of low frequency heart rate variability as an index of sympathetic cardiac tone: a review with emphasis on a reanalysis of previous studies. Psychophysiology. 2013;50(5):477–87. doi: 10.1111/psyp.12027 23445494

[27] Geisler FCM, Kubiak T, Siewert K, Weber H. Cardiac vagal tone is associated with social engagement and self-regulation. Biol Psychol. 2013;93(2):279–86. doi: 10.1016/j.biopsycho.2013.02.013 23466587

[28] Williams DP, Cash C, Rankin C, Bernardi A, Koenig J, Thayer JF. Resting heart rate variability predicts self-reported difficulties in emotion regulation: a focus on different facets of emotion regulation. Front Psychol. 2015;6:261. doi: 10.3389/fpsyg.2015.00261 25806017 PMC4354240

[29] Neumann SA, Sollers JJ 3rd, Thayer JF, Waldstein SR. Alexithymia predicts attenuated autonomic reactivity, but prolonged recovery to anger recall in young women. Int J Psychophysiol. 2004;53(3):183–95. doi: 10.1016/j.ijpsycho.2004.03.008 15246672

[30] Kosonogov V, De Zorzi L, Honoré J, Martínez-Velázquez ES, Nandrino J-L, Martinez-Selva JM, et al. Facial thermal variations: A new marker of emotional arousal. PLoS One. 2017;12(9):e0183592. doi: 10.1371/journal.pone.0183592 28922392 PMC5603162

[31] Rodríguez Medina DA, Domínguez Trejo B, Cortés Esteban P, Cruz Albarrán IA, Morales Hernández LA, Leija Alva G. Biopsychosocial assessment of pain with thermal imaging of emotional facial expression in breast cancer survivors. Medicines (Basel). 2018;5(2):30. doi: 10.3390/medicines5020030 29601485 PMC6023480

[32] Russell JA. A circumplex model of affect. Journal of Personality and Social Psychology. 1980;39(6):1161–78. doi: 10.1037/h0077714

[33] Pavlidis I, Tsiamyrtzis P, Shastri D, Wesley A, Zhou Y, Lindner P, et al. Fast by nature - how stress patterns define human experience and performance in dexterous tasks. Sci Rep. 2012;2:305. doi: 10.1038/srep00305 22396852 PMC3294268

[34] Hahn AC, Whitehead RD, Albrecht M, Lefevre CE, Perrett DI. Hot or not? Thermal reactions to social contact. Biol Lett. 2012;8(5):864–7. doi: 10.1098/rsbl.2012.0338 22647931 PMC3440979

[35] Ioannou S, Gallese V, Merla A. Thermal infrared imaging in psychophysiology: potentialities and limits. Psychophysiol. 2014;51:951–63. doi: 10.1111/psyp.12243 PMC4286005 24961292

[36] Goerlich KS. The multifaceted nature of Alexithymia - A neuroscientific perspective. Front Psychol. 2018;9:1614. doi: 10.3389/fpsyg.2018.01614 30210420 PMC6124373

[37] Snellen H. Probebuchstaben zur Bestimmung der Sehschärfe. Utrecht: Van de Weijer; 1862.

[38] Bagby RM, Parker JD, Taylor GJ. The twenty-item Toronto Alexithymia Scale--I. Item selection and cross-validation of the factor structure. J Psychosom Res. 1994;38(1):23–32. doi: 10.1016/0022-3999(94)90005-1 8126686

[39] Bailey PE, Henry JD. Alexithymia, somatization and negative affect in a community sample. Psychiatry Res. 2007;150(1):13–20. doi: 10.1016/j.psychres.2006.05.024 17258817

[40] Loas G, Corcos M, Stephan P, Pellet J, Bizouard P, Venisse JL, et al. Factorial structure of the 20-item Toronto Alexithymia Scale: confirmatory factorial analyses in nonclinical and clinical samples. J Psychosom Res. 2001;50(5):255–61. doi: 10.1016/s0022-3999(01)00197-0 11399282

[41] Deborde A-S, Berthoz S, Wallier JM, Fermanian J, Falissard B, Jeammet P, et al. The Bermond-Vorst Alexithymia Questionnaire cutoff scores: a study in eating-disordered and control subjects. Psychopathology. 2008;41(1):43–9. doi: 10.1159/000109955 17952021

[42] Taylor GJ, Bagby RM, Parker JDA. The 20-Item Toronto Alexithymia Scale. IV. Reliability and factorial validity in different languages and cultures. J Psychosom Res. 2003;55:277–83. doi: 10.1016/s0022-3999(02)00601-3 12932803

[43] Bermond B, Clayton K, Liberova A, Luminet O, Maruszewski T, Ricci Bitti PE, et al. A cognitive and an affective dimension of alexithymia in six languages and seven populations. Cognition and Emotion. 2007;21(5):1125–36. doi: 10.1080/02699930601056989

[44] Bagby RM, Quilty LC, Taylor GJ, Grabe HJ, Luminet O, Verissimo R, et al. Are there subtypes of alexithymia? Personality and Individual Differences. 2009;47:413–8. doi: 10.1016/j.paid.2009.04.012

[45] Laborde S, Mosley E, Thayer JF. Heart rate variability and cardiac vagal tone in psychophysiological research - recommendations for experiment planning, data analysis, and data reporting. Front Psychol. 2017;8:213. doi: 10.3389/fpsyg.2017.00213 28265249 PMC5316555

[46] Malik M, Bigger JT, Camm AJ, Kleiger RE, Malliani A, Moss AJ, et al. Heart rate variability: Standards of measurement, physiological interpretation, and clinical use. European Heart Journal. 1996;17(3):354–81. doi: 10.1093/oxfordjournals.eurheartj.a014868 8737210

[47] Davis MH. Measuring individual differences in empathy: Evidence for a multidimensional approach. Journal of Personality and Social Psychology. 1983;44(1):113–26. doi: 10.1037/0022-3514.44.1.113

[48] Liebowitz MR. Social phobia. Mod Probl Pharmacopsychiatry. 1987;22:141–73. doi: 10.1159/000414022 2885745

[49] Spielberger CD. Inventaire d’anxiété État-Trait: Forme Y. ECPA, les Éditions du centre de psychologie appliquée. 1993.

[50] Beck AT, Steer RA, Brown GK. Beck depression inventory-II. San Antonio. 1996;78:490–8.

[51] Lang PJ, Bradley MM, Cuthbert BN. International affective picture system (IAPS): affective ratings of pictures and instruction manual. A-8. Gainesville, FL: University of Florida; 2008.

[52] Groen Y, Wijers AA, Tucha O, Althaus M. Are there sex differences in ERPs related to processing empathy-evoking pictures?. Neuropsychologia. 2013;51(1):142–55. doi: 10.1016/j.neuropsychologia.2012.11.012 23174404

[53] Proverbio AM, Adorni R, Zani A, Trestianu L. Sex differences in the brain response to affective scenes with or without humans. Neuropsychologia. 2009;47(12):2374–88. doi: 10.1016/j.neuropsychologia.2008.10.030 19061906

[54] Steketee J. Spectral emissivity of skin and pericardium. Phys Med Biol. 1973;18(5):686–94. doi: 10.1088/0031-9155/18/5/307 4758213

[55] Ioannou S, Morris P, Terry S, Baker M, Gallese V, Reddy V. Sympathy Crying: Insights from Infrared Thermal Imaging on a Female Sample. PLoS One. 2016;11(10):e0162749. doi: 10.1371/journal.pone.0162749 27716801 PMC5055358

[56] Bradley MM, Lang PJ. Measuring emotion: the Self-Assessment Manikin and the Semantic Differential. J Behav Ther Exp Psychiatry. 1994;25(1):49–59. doi: 10.1016/0005-7916(94)90063-9 7962581

[57] Nhan BR, Chau T. Classifying affective states using thermal infrared imaging of the human face. IEEE Trans Biomed Eng. 2010;57(4):979–87. doi: 10.1109/TBME.2009.2035926 19923040

[58] Bradley MM, Lang PJ. The International Affective Picture System (IAPS) in the Study of Emotion and Attention. Handbook of Emotion Elicitation and Assessment. Oxford University PressNew York, NY; 2007. p. 29–46. doi: 10.1093/oso/9780195169157.003.0003

[59] D’Hondt F, Lassonde M, Collignon O, Dubarry A-S, Robert M, Rigoulot S, et al. Early brain-body impact of emotional arousal. Front Hum Neurosci. 2010;4:33. doi: 10.3389/fnhum.2010.00033 20428514 PMC2859881

[60] Cacioppo JT, Berntson GG, Larsen JT, Poehlmann KM, Ito TA. Handbook of emotions. 2000. 173–91.

[61] Kreibig SD. Autonomic nervous system activity in emotion: a review. Biol Psychol. 2010;84(3):394–421. doi: 10.1016/j.biopsycho.2010.03.010 20371374

[62] Maxwell SE, Delaney HD. Designing experiments and analyzing data: A model comparison perspective. 2nd ed. Mahwah, NJ, US: Lawrence Erlbaum Associates Publishers; 2004.

[63] Rosenthal R. Contrast analysis: focused comparisons in the analysis of variance. Cambridge: Cambridge University Press; 1985.

[64] Faul F, Erdfelder E, Lang A-G, Buchner A. G*Power 3: a flexible statistical power analysis program for the social, behavioral, and biomedical sciences. Behav Res Methods. 2007;39(2):175–91. doi: 10.3758/bf03193146 17695343

[65] Bakdash JZ, Marusich LR. Repeated measures correlation. Front Psychol. 2017;8:456. doi: 10.3389/fpsyg.2017.00456 28439244 PMC5383908

[66] Cohen J. Statistical power analysis for the behavioral sciences. 2nd ed. New York: Routledge; doi: 10.4324/9780203771587

[67] Fukunishi I, Kikuchi M, Wogan J, Takubo M. Secondary alexithymia as a state reaction in panic disorder and social phobia. Compr Psychiatry. 1997;38(3):166–70. doi: 10.1016/s0010-440x(97)90070-5 9154373

[68] Grynberg D, Chang B, Corneille O, Maurage P, Vermeulen N, Berthoz S, et al. Alexithymia and the processing of emotional facial expressions (EFEs): systematic review, unanswered questions and further perspectives. PLoS One. 2012;7(8):e42429. doi: 10.1371/journal.pone.0042429 22927931 PMC3426527

[69] Hesse C, Floyd K. Affectionate experience mediates the effects of alexithymia on mental health and interpersonal relationships. Journal of Social and Personal Relationships. 2008;25(5):793–810. doi: 10.1177/0265407508096696

[70] Pisani S, Murphy J, Conway J, Millgate E, Catmur C, Bird G. The relationship between alexithymia and theory of mind: A systematic review. Neurosci Biobehav Rev. 2021;131:497–524. doi: 10.1016/j.neubiorev.2021.09.036 34599917

[71] Hoffmann F, Banzhaf C, Kanske P, Gärtner M, Bermpohl F, Singer T. Empathy in depression: Egocentric and altercentric biases and the role of alexithymia. J Affect Disord. 2016;199:23–9. doi: 10.1016/j.jad.2016.03.007 27060429

[72] Luminet O, Nielson KA, Ridout N. Cognitive-emotional processing in alexithymia: an integrative review. Cogn Emot. 2021;35(3):449–87. doi: 10.1080/02699931.2021.1908231 33787442

[73] Luminet O, Rimé B, Bagby RM, Taylor G. A multimodal investigation of emotional responding in alexithymia. Cognition & Emotion. 2004;18(6):741–66. doi: 10.1080/02699930341000275

[74] Cuve HC, Castiello S, Shiferaw B, Ichijo E, Catmur C, Bird G. Alexithymia explains atypical spatiotemporal dynamics of eye gaze in autism. Cognition. 2021;212:104710. doi: 10.1016/j.cognition.2021.104710 33862441

[75] Bird G, Silani G, Brindley R, White S, Frith U, Singer T. Empathic brain responses in insula are modulated by levels of alexithymia but not autism. Brain. 2010;133(Pt 5):1515–25. doi: 10.1093/brain/awq060 20371509 PMC2859151

[76] Shaffer F, McCraty R, Zerr CL. A healthy heart is not a metronome: an integrative review of the heart’s anatomy and heart rate variability. Front Psychol. 2014;5:1040. doi: 10.3389/fpsyg.2014.01040 25324790 PMC4179748

[77] Forte G, Favieri F, Oliha EO, Marotta A, Casagrande M. Anxiety and attentional processes: the role of resting heart rate variability. Brain Sci. 2021;11(4):480. doi: 10.3390/brainsci11040480 33918848 PMC8070415

[78] Park G, Thayer JF. From the heart to the mind: cardiac vagal tone modulates top-down and bottom-up visual perception and attention to emotional stimuli. Frontiers in Psychology. 2014;5. doi: 10.3389/fpsyg.2014.00278 PMC4013470 24817853

[79] Porges SW. Polyvagal theory: a science of safety. Frontiers in Integrative Neuroscience. 2022;16. 10.3389/fnint.2022.871227 PMC9131189 35645742

[80] Merla A, Romani GL. Thermal signatures of emotional arousal: a functional infrared imaging study. Annu Int Conf IEEE Eng Med Biol Soc. 2007;2007:247–9. doi: 10.1109/IEMBS.2007.4352270 18001936

[81] Ioannou S, Ebisch S, Aureli T, Bafunno D, Ioannides HA, Cardone D, et al. The autonomic signature of guilt in children: a thermal infrared imaging study. PLoS One. 2013;8(11):e79440. doi: 10.1371/journal.pone.0079440 24260220 PMC3834185

[82] Salazar-López E, Domínguez E, Juárez Ramos V, de la Fuente J, Meins A, Iborra O, et al. The mental and subjective skin: Emotion, empathy, feelings and thermography. Conscious Cogn. 2015;34:149–62. doi: 10.1016/j.concog.2015.04.003 25955182

[83] Kozubal M, Szuster A, Wielgopolan A. Emotional regulation strategies in daily life: the intensity of emotions and regulation choice. Front Psychol. 2023;14:1218694. doi: 10.3389/fpsyg.2023.1218694 37645071 PMC10460911

[84] Chen J, Xu T, Jing J, Chan RCK. Alexithymia and emotional regulation: A cluster analytical approach. BMC Psychiatry. 2011;11:33. doi: 10.1186/1471-244X-11-33 21345180 PMC3050802

[85] Laloyaux J, Fantini C, Lemaire M, Luminet O, Larøi F. Evidence of contrasting patterns for suppression and reappraisal emotion regulation strategies in Alexithymia. J Nerv Ment Dis. 2015;203(9):709–17. doi: 10.1097/NMD.0000000000000353 26252826

[86] Nozawa A, Tacano M. Correlation analysis on alpha attenuation and nasal skin temperature. J Stat Mech. 2009;2009(01):P01007. doi: 10.1088/1742-5468/2009/01/p01007

[87] Panasiti MS, Cardone D, Pavone EF, Mancini A, Merla A, Aglioti SM. Thermal signatures of voluntary deception in ecological conditions. Sci Rep. 2016;6:35174. doi: 10.1038/srep35174 27734927 PMC5062078

[88] Ventura-Bort C, Wendt J, Weymar M. The role of interoceptive sensibility and emotional conceptualization for the experience of emotions. Front Psychol. 2021;12:712418. doi: 10.3389/fpsyg.2021.712418 34867591 PMC8636600

[89] Matsumoto N, Watson LA, Fujino M, Ito Y, Kobayashi M. Subjective judgments on direct and generative retrieval of autobiographical memory: The role of interoceptive sensibility and emotion. Mem Cognit. 2022;50(8):1644–63. doi: 10.3758/s13421-022-01280-8 35294741

